# Extrauterine Listeriosis in the Gravid Mouse Influences Embryonic Growth and Development

**DOI:** 10.1371/journal.pone.0072601

**Published:** 2013-08-14

**Authors:** M. Mitsu Suyemoto, Terri S. Hamrick, Patricia A. Spears, John R. Horton, Ida M. Washington, Edward A. Havell, Luke B. Borst, Paul E. Orndorff

**Affiliations:** 1 Department of Population Health and Pathobiology, College of Veterinary Medicine, North Carolina State University, Raleigh, North Carolina, United States of America; 2 Department of Biomedical Sciences, College of Veterinary Medicine, North Carolina State University, Raleigh, North Carolina, United States of America; East Carolina University School of Medicine, United States of America

## Abstract

Gravid mice and other rodents inoculated with *Listeria monocytogenes* typically fail to clear an intrauterine infection and either succumb or expel their intrauterine contents. We took advantage of this property to investigate the effects of an extrauterine infection on parameters of pregnancy success. Pregnant mice were selected for our study if they showed no clinical signs of listeriosis following oral inoculation at 7.5 gestational days (gd), and had no detectable intrauterine colony forming units (cfu) at near term (18.5 gd). The range of oral doses employed was 10^6^-10^8^ cfu per mouse for two listerial serotype strains (4nonb and 1/2a). At all doses, inoculation resulted in a decrease in average near-term (18.5 gd) fetal weight per litter compared to sham inoculated controls. Additionally, embryonic death (indicated by intrauterine resorptions) was exhibited by some inoculated mice but was absent in all sham inoculated animals. In parallel experiments designed to detect possible loss of placental function, gravid uteruses were examined histopathologically and microbiologically 96 h after oral inoculation. Placental lesions were associated with high (> 10^6^), but not low (< 10^2^) or absent intrauterine cfu. In vitro, mouse embryonic trophoblasts were indistinguishable from mouse enterocytes in terms of their sensitivity to listerial exposure. A model consistent with our observations is one in which products (host or bacterial) generated during an acute infection enter embryos transplacentally and influences embryonic survival and slows normal growth in utero.

## Introduction

Foodborne listeriae that escape the intestinal lumen, enter the circulation and multiply in the liver and spleen [1]. Microorganisms that are not quickly eliminated, re-enter the circulation and infect other vital organs [2]. In these instances, nongravid hosts typically experience neurological complications; whereas, gravid individuals have complications of pregnancy [3]. Ample experimental evidence from rodent models indicates that a gravid individual is the equal of her nonpregnant counterpart in containing the initial infection to the liver and spleen, but the disease takes a devastating turn when escaping listeriae colonize the gravid uterus [4–6]. This is due to the uterus serving as an immunologically privileged nidus that perpetuates infection [7] from the time of blastocyst implantation and subsequently through embryonic development [8]. The effect of an extrauterine infection on pregnancy success is unclear even though, in rodents, the uterus is relatively resistant to infection compared to the liver and spleen [4,5,8,9]. In orally inoculated gravid mice, the rate of intrauterine infection is *ca*. half that of the liver and spleen [5].

Mice have an 18.5-20 gestation day (gd) period and development takes place most rapidly between 5.5 and 9.5 days. During this period the embryo is highly susceptible to toxic hematogenously borne agents [10] and can serve as a sensitive indicator of the effects of an extrauterine listerial infection. This is particularly true in animals with hemochorial placentation (humans, rodents [11]) where blood borne infectious agents, and their products, come into direct contact with embryonic trophoblastic cells [12]. In mice orally inoculated at 7.5 gd, the intrauterine infection rate (determined *ca.* 96 h post inoculation) is indistinguishable from the death rate in animals inoculated in parallel and allowed to proceed to term [5]. Similarly, in other rodents, there is no indication that an intrauterine infection can be effectively cleared without expulsion of the intrauterine contents [7]. These results imply that inoculated gravid animals that lack signs of disease during gestation are those most likely to have experienced an extrauterine infection.

Herein, we report that orally inoculated gravid animals that showed no signs of infection, or any evidence of intrauterine colonization produced near term fetuses of lower average weight and had an increased incidence of embryo resorptions. We found no histological evidence of placental damage during acute extrauterine infections and no evidence that mouse embryonic trophoblasts were unusually sensitive to listerial exposure in vitro.

## Materials and Methods

### Ethics Statement

This study was carried out in strict accordance with the recommendations in the Guide for the Care and Use of Laboratory Animals of the National Institutes of Health. The study was approved by the North Carolina State University Institutional Animal Care and Use Committee (Assurance Number: A3331-01).

### Bacterial strains and growth conditions

The mouse oral virulent *L. monocytogenes* serotype 4nonb strains F6214-1, PAS351 [5], and F6212 (referred to as G9599 by Pine et al. [13]) were employed as well as the serotype 1/2a strain 10403S [14]. PAS351 is a genetically marked version of F6214-1 employed in inoculations in a 1:1 mixture. In some experiments, a 1:1 mixture of F6214-1 and PAS351 was used in order to detect possible infectious bottlenecks [8]. This precaution proved unnecessary and total listerial numbers are presented unless otherwise indicated. Bacteria were propagated at 37° C, in brain heart infusion (BHI) broth, or BHI broth supplemented with 1.5% agar (Difco). Broth cultures were grown overnight with shaking. The resulting stationary phase culture was subcultured and grown with shaking to logarithmic phase (OD_600_ 0.3-0.6)**.**

### Timed pregnant mice

CD-1 mouse pairings were carried out by housing one male with three females in the evening. Pregnant mice were identified by the presence of a postcoital vaginal plug the following morning. Gestational days (gd) were established based on an initial 0.5 gd determination the morning following mating. Sets of nonpregnant and 6.5 gd pregnant mice were inoculated either orally or intragastrically with the doses and strains of *L. monocytogenes* specified in the Results section.

### Inoculations and organ infectivity

Bacteria were harvested by centrifugation and the pellet resuspended in phosphate buffered saline (PBS). Colony forming units [cfu] were determined as previously described [5]. Mice were orally inoculated with a 20 µl volume after depriving them of water for 2 h [15] or via gavage as previously described [5]. Organ infectivity, including intrauterine infection, was determined at various time points using methods previously described [15].

### Histological methods

In those instances where the gravid uteruses were examined histologically, one uterine horn was fixed in 10% neutral buffered formalin, processed into paraffin, sectioned and stained for microscopic examination, as previously described [5]. Histological assessments were conducted by a veterinary pathologist (author LB) blinded to infection status. Lesions, when present, were confined to the placenta with no histologic changes noted in the embryos. For each mouse, histologic evidence of inflammation and infection were scored for 5 embryo/placental units as follows: Inflammatory changes consisting of neutrophilic infiltration into the placenta, fibrin deposition, and necrosis were subjectively scored for severity (normal = 0, mild =1, moderate =2 and severe =3). Intrauterine infection was assessed separately using Gram-stained sections and the presence or absence of listerial organisms was scored as 0 (absent) or 1 (present) in 5 embryo/placental units. Aggregate uterine inflammation and infection scores for each mouse were created by summing scores of the 5 embryo/placental units.

### Cell lines and cell culture methods

The mouse enterocyte cell line, MODE K [16], was propagated as a monolayer under conditions previously described [5]. The mouse trophoblastic cell line, SM10 [17,18], was propagated as a monolayer in RPMI 1640 medium containing 5% FBS, 1 mM sodium pyruvate, 50 µM 2-mercaptoethanol.

### Listerial plaque assays on MODE K and SM10 monolayers

Bacteria were suspended in 1.0 ml of Dulbecco’s Modified Eagle Medium (DMEM) and 5% FBS at various concentrations were added to monolayers in 6 well (35 mM) tissue culture plates for 10 min. Subsequent listerial removal and incubation in the presence of gentamicin sulfate (5 µg/ml) was as described in Hamrick et al. [5]. Plaques were detected and enumerated as previously described [19].

### Statistical analysis

Standard deviation of the mean was calculated with the aid of the Microsoft Excel STDEV function. Standard error was calculated by dividing the standard deviation by the square root of the number of samples. Significant differences between means were determined using Student’s t test with the aid of the Excel’s TTEST function and Fisher’s exact test with the aid of GraphPad Prism® version 5.04. Linear regression analysis was done using Excel’s LINEST, FTEST, STEYX functions. The alpha error probability threshold was P <0.05.

## Results

### The influence of extrauterine listeriae on pregnancy

Gravid animals, gavage inoculated at 7.5 gd with varying doses of two different listerial serogroup strains, were sacrificed at 18.5 gd (near term). Among the animals that showed no clinical signs of infection were those that were visibly gravid by 18.5 gd. Other inoculated animals that showed no clinical signs of infection were found, at necropsy, to have never been pregnant, or in 2 cases, had resorbed all their embryos. Homogenates of the entire uterus and intrauterine contents of all mice (excluding nonpregnant individuals) yielded no detectable listeriae. Mice that displayed clinical signs of infection (e.g., piloerection, huddling) either died or exhibited hemorrhaging and expulsion before the 18.5 gd near-term time point. In gravid asymptomatic mice, we noted (Table **1**) that average litter size was not influenced by listerial inoculation at any dose or strain employed. However, other parameters of fetal well-being showed significant differences between the sham and listeriae inoculated mice. In particular, gravid mice inoculated with strain F6212 had litters with significantly lower average fetal near-term weights than sham inoculated mice--regardless of dose. Gravid mice inoculated with strain 10403S, although showing a similar reduction in average pup weight per litter, had means that were not distinguishably different. If the average weight of all pups was considered, rather than the average weight per litter, then inoculation with either strain resulted in pups of significantly lower birthweight.

**Table 1:** Pregnancy outcomes from gravid mice showing no signs of listeriosis and culture-negative intrauterine contents.

Strain inoculated	Dose per mouse^a^	Number of mice examined	Mice with viable fetuses	Average litter size^b^	Total number of viable fetuses	Average viable fetal weight (g)	Average viable fetal weight (g) per litter^c^	Number of mice with resorbed embryos^d^	Average resorptions per gravid mouse	Average resorptions per viable fetus
Sham	0	5	5	14.2 ± 1.9	71	1.43 ± 0.15	1.43 ± 0.07	0	0	0
F6212	1 x 10^6^	3	3	14.0 ± 3.6	42	**1.34 ± 0.08**	**1.34 ± 0.06**	2	1	0.07
F6212	1 x 10^7^	2	1	15.0 ± (0)	15	**1.28 ± 0.05**	1.29 ± (0)	1	4	**0.53**
F6212	1 x 10^8^	4	4	14.5 ± 2.4	58	**1.31 ± 0.12**	**1.31 ± 0.00**	0	0	0
10403S	1 x 10^7^	5	4	13.3 ± 1.5	53	**1.33 ± 0.13**	1.34 ± 0.11	1	2.6	**0.25**

Average values significantly different from the sham inoculated group are in bold.

a Mice were intragastrically inoculated the colony forming units (cfu) denoted, or sham inoculated with PBS at 6.5 gd as described in the text.

b Only litters with viable fetuses are considered. Only viable fetuses were counted in litter size determinations. ± denotes standard deviation (SD). A parenthetical SD indicates that a single litter was examined and consequently could not be included in the statistical analysis.

c Calculations were made by summing the average fetal weight per litter and diving by the number of gravid animals in each group. A parenthetical SD value indicates that a single litter was examined and consequently could not be included in the statistical analysis.

d One mouse inoculated with F6212 at 1 x 10^7^ cfu and one mouse inoculated with 10403S had only resorbed embryos, no viable ones.

In addition to the lower fetal weights, embryo resorption was only observed in inoculated animals. At some inoculation doses, resorption rates, calculated as resorptions per viable fetus, occurred at rates significantly higher than in sham inoculated animals (refer to Table 1). Resorption site morphology and size indicated that the resorptions occurred within 48-72 h of inoculation.

### Uterine histological examination 96 h following oral inoculation at 6.5 gd

**Figure 1:**
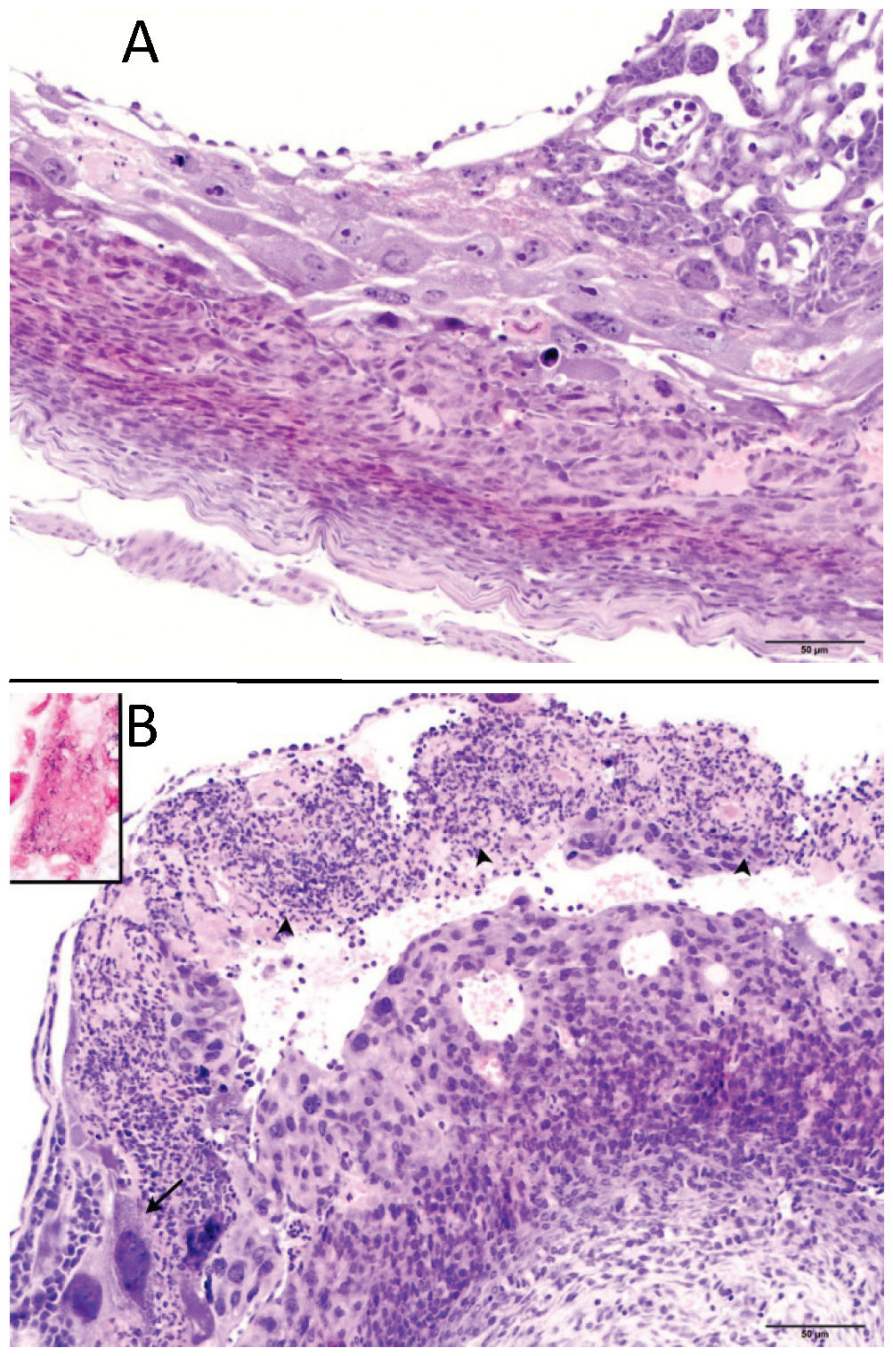
Histological examination of conceptuses from mice with intrauterine and extrauterine infections. Hematoxylin/eosin stained sections of the conceptuses from two of five mice identified in Table 2 are shown. (A) Uterus and placenta (200x magnification) of mouse 2 with no detected uterine cfu: no lesions were noted. (B) Uterus and placenta (200x magnification) from mouse 7, showing > 10^6^ uterine listerial cfu. The arrow heads denote inflammation and necrosis indicated by the degenerative neutrophils mixed with cellular debris and fibrin that have focally effaced the chorionic plate. The arrow identifies an infected giant trophoblast that has intracellular gram positive rods (see Gram-stained inset: 1000x).

One uterine horn from each of 5 orally inoculated gravid mice (each receiving *ca.* 2 x 10^8^ cfu of strain F6214-1) was examined histologically. At the time of sacrifice 96 h later, all five mice exhibited detectable cfu in their colon contents, spleen, and (with one exception) liver (Table **2**). Two of the mice had intrauterine listerial cfu detected in the uterine horn taken for microbiological analysis. Evaluation of the horn taken for histologic examination revealed no lesions in the placenta and embryos from mice that showed no cfu in their opposite uterine horn (e.g., mouse 2, Figure **1A**). However, lesions consisting of suppurative inflammation and necrosis with intralesional bacilli were readily detected in the animal with the highest intrauterine listerial cfu (mouse 7, Figure **1B**). The mouse having comparatively few uterine cfu (mouse 5, Table 2) appeared histologically normal using our scoring system and indistinguishable from mice having no detectable cfu.

**Table 2:** Listerial cfu in mouse tissues 96 h post inoculation^a^

		cfu recovered from^b^:			Histopathology scores^c^	
Mouse reference number	Uterus	Spleen	Liver	Colon	Inflammation	Infection
2	0	1.15 x 10^3^	5.00 x 10^1^	5.00 x 10^1^	0	0
3	0	7.80 x 10^3^	5.45 x 10^3^	3.50 x 10^2^	0	0
5	7.00 x 10^2^	2.50 x 10^5^	1.80 x 10^5^	7.50 x 10^2^	0	0
6	0	9.85 x 10^3^	0	3.00 x 10^2^	0	0
7	5.90 x 10^6^	4.90 x 10^6^	1.90 x 10^6^	3.75 x 10^4^	6	3

^a^ Nine mice at 6.5 gestational days were orally inoculated with *ca.* 2 x 10^8^ cfu of strain F6214-1. Four mice were later found not to be pregnant. Results from the remaining 5 mice are tabulated.

b Cfu are tabulated per organ (limit of detection 50 cfu per organ).

c Sum of scores for 5 embryos obtained from each mouse as determined in a blinded study described in the text.

### Trophoblast sensitivity to listerial exposure in vitro

**Figure 2:**
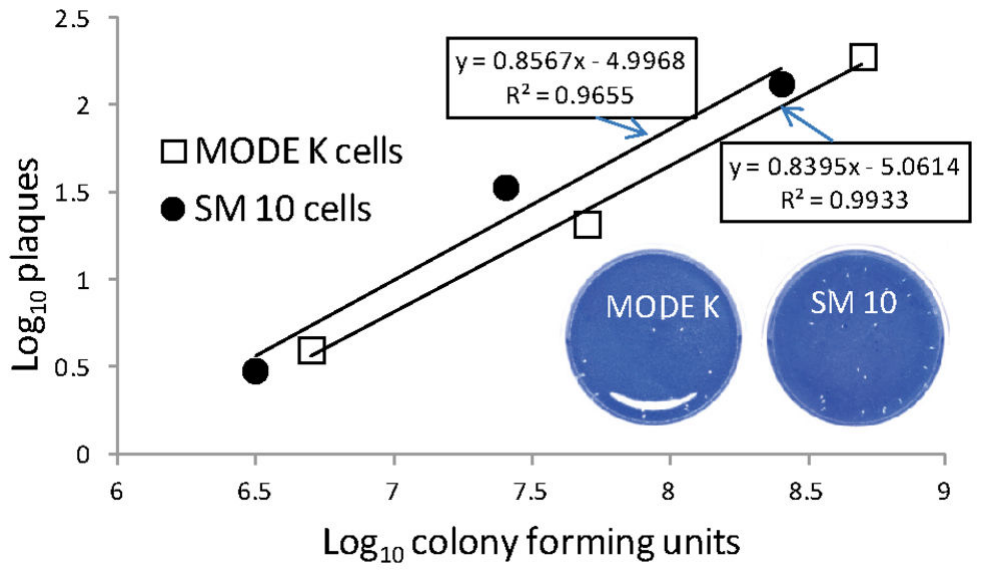
Linear regression analysis of plaquing efficiency supported by MODE K (enterocyte) and SM10 (trophoblast) monolayers. Listerial strain F6214-1 was employed in the comparison. The inset shows representative monolayers with listerial plaques on each cell type for comparison. MODE K designates our mouse enterocyte cell line and SM10 our mouse trophoblast cell line.

Mouse embryonic trophoblastic cells (SM10) were compared to mouse enterocyte cells (MODE K) for their ability to provide a protective environment for listerial growth over a wide range of exogenous listerial doses. We reasoned that, if listerial exposure was detrimental to the host cell’s ability to support growth, then it would be reflected most quantitatively in a loss of plaquing efficiency due to a loss of the cell’s ability to exclude gentamicin. Linear regression analysis revealed that MODE K and SM10 monolayers were indistinguishable (F test) in their ability to effectively support gentamicin protected intracellular growth over a wide exposure range with no demonstrable loss of plaquing efficiency (R^2^ > 0.95) with dose (Figure **2**) or loss of monolayer integrity (Figure 2 inset).

## Discussion

Most descriptions of gestational listeriosis concentrate on fetal loss or the neonatal complications attendant with intrauterine colonization. Very little is known about uncomplicated listeriosis in which the infection remains extrauterine. Nevertheless, the effects could be profound, especially early in gestation where 50% of spontaneous abortions before 20 gestational weeks (i.e., miscarriages—the most common form of pregnancy loss [20]) are of uncertain etiology [21]. In the present study, we examined populations of clinically normal gravid mice that had been intragastrically inoculated with *L. monocytogenes* to see if there was an influence of an extrauterine infection on pregnancy success. Untoward effects were readily detected. The effects could not be attributed simply to placental damage or to or to a hypersensitivity of trophoblasts to listerial exposure. A model most consistent with our observations is one in which detrimental products (host or bacterial) generated during an acute subclinical infection enter the embryo through a fully functioning placenta.

Examination of inoculated gravid mice revealed that litter size was not detectably influenced. Since litter size influences birth weight, the constant litter size allowed us to directly compare the average near-term weights of pups from individual mothers (i.e., each n equals a gravid animal with pups of an average birth weight) as well as in aggregate (i.e., each n equals an individual pup with a specific birth weight). Regardless of the type of analysis employed, there was a striking reduction in near-term birthweight associated with inoculation of listerial strain F6212. Strain 10403S exhibited an influence on birth weight if pups were compared in aggregate but not if compared by individual mother. In contrast to the serogroup 4 strains we employed (F6212, F6214-1), the serogroup 1/2a strain employed (10403S) is regarded as attenuated in mice via the oral route because of its failure to effectively translocate from the intestinal lumen [5,22,23]. Any influence on development would thus be attributable to a luminal translocating factor. Alternatively, our prior studies indicate that strain 10403S exhibits a degree of luminal escape, especially in pregnant animals [5]. This escape may be sufficient to produce the effects witnessed.

An embryo resorption rate was readily calculated in the inoculated animals but no resorptions occurred in the sham inoculated animals. Resorptions are analytically valuable because the size and appearance of the resorption site allows an accurate estimate of the time the embryo stopped developing [24]. In all cases herein noted, resorption occurred within 48-72 h post inoculation (a period corresponding to the acute listerial infection [5]).

When employing graded doses (10^6^-10^9^ cfu) of strain F6212, we found that even at the lowest dose employed (*ca.* 0.01 LD_50_ in gravid animals [5]) an influence on mean fetal weight and resorption rate could be readily detected. However, there was no trend toward progressively lower birth weights and increased resorption rates with dose. The lack of a trend is likely due to our requirement that all enrolled animals show no disease signs. That is, some animals clearly had to resist larger inoculating doses than others, but all had to share the common trait of doing this successfully.

Mechanistically, resorption and low birthweight could be caused by a loss of placental function or by the structure’s effective transfer of compounds produced (or induced) by circulating listeriae. We found no histologic evidence of placental damage in the absence of listerial intrauterine colonization. Similarly, we found no evidence that listerial exposure impaired mouse trophoblast monolayer function or appearance when compared to a control mouse enterocyte monolayer. These results suggest that placental function is not easily lost by simple listerial exposure--such as during a transient bacteremia.

Our present model is that a listerial infection generates products (bacterial or host derived) that cross the placenta slow normal embryonic growth (producing lower birthweights) and less frequently, produce embryonic death. In mice, the products of other infectious agents associated with resorptions and fetal growth impairment (in particular, periodontal member of the *Campylobacter* genus [25,26]) have been proposed to act through lipopolysaccharide (LPS)-stimulated TNF-α production to produce resorptions [27]. Whereas these infections are typically chronic and involve intrauterine colonization, it is possible that, in the gram positive listeriae, wall teichoic acid and lipoteichoic acid may act similarly to LPS. Also, it is well known that TNF-α production is associated with listerial infection in mice [28]. In a recent report on the role of T regulatory cells in murine fetal wastage during listerial infection by Rowe et al. [29], the authors note an extrauterine effect on fetal resorptions following intravenous inoculation of low doses of strain 10403S at midgestation (10.5 gd), that are (in part) similar to the effects reported here. The authors suggest an immune mediated detrimental effect on fetal viability. Further studies, with well defined host and listerial mutants (e.g., teichoic acid mutants), may identify and better define the factors involved.
